# Comparison between indigenous and Western postnatal care practices in Mopani District, Limpopo Province, South Africa

**DOI:** 10.4102/curationis.v38i1.1252

**Published:** 2015-05-28

**Authors:** Roinah N. Ngunyulu, Fhumulani M. Mulaudzi, Mmapheko D. Peu

**Affiliations:** 1 Department of Nursing Sciences, University of Pretoria, South Africa

## Abstract

**Background:**

Postnatal care begins immediately after the expulsion of the placenta and continues for six to eight weeks post-delivery. High standard of care is required during the postnatal period because mothers and babies are at risk and vulnerable to complications related to postpartum haemorrhage and infections. Midwives and traditional birth attendants are responsible for the provision of postnatal care in different settings, such as clinics and hospitals, and homes.

**Methods:**

A qualitative, exploratory, descriptive and contextual research approach was followed in this study. Unstructured interviews were conducted with the traditional birth attendants. An integrated literature review was conducted to identify the Western postnatal care practices. Tesch's process was followed during data analysis.

**Findings:**

The following main categories were identified: similarities between indigenous and Western postnatal care practices, and differences between indigenous and Western postnatal care practices. Based on these findings, training of midwives and traditional birth attendants was recommended in order to empower them with knowledge and skills regarding the indigenous and Western postnatal care practices.

**Conclusions:**

It is evident that some indigenous postnatal care practices have adverse effects on the health of postnatal women and their newborn infants, but these are unknown to the traditional birth attendants. The employment of indigenous postnatal care practices by the traditional birth attendants is also influenced by their cultural beliefs, norms, values and attitudes. Therefore, there is an urgent need to train midwives and traditional birth attendants regarding the indigenous and Western postnatal care to improve the health of postnatal women and their babies.

## Introduction

### Background

This article reports an important part of the study conducted during the principal investigator's Master's degree, entitled: *Indigenous practices regarding postnatal care at Sikhunyani village, Mopani District, Limpopo Province of South Africa.* The study had two objectives, which were to explore and describe the indigenous practices regarding postnatal care, and to compare the similarities and differences between the indigenous and Western postnatal care practices.

South Africa as a developing country is challenged with increasing rates of maternal and neonatal mortality, despite the fact that the recommended reduction strategies are in place. According to the World Health Organisation's (WHO 2013) maternal mortality statistics from 1990 to 2013, South Africa stands at 140 deaths per 100 000 live birth, as compared to Nigeria that has 560 deaths per 100 000 live births. Globally from 1990 to 2013, the maternal mortality ratio declined by 45% from 380 deaths per 100 000 live births to 210 deaths per 100 000 live births, according to UN inter-agency estimates. However, there are still unacceptably high disparities between the developed and developing countries regarding maternal mortality rates. For example, Australia stands at 6 deaths per 100 000 live births, whereas Central African Republic has 880 deaths per 100 000 live births. This shows that all maternal deaths throughout the world, including those in poor countries, can be prevented as seen in developed countries such as Australia (WHO 2013). Despite the availability and accessibility of maternal and child health services in South Africa, 50% of women still consult traditional birth attendants as their first choice during pregnancy, labour, delivery and postnatal care (Ngomane & Mulaudzi 2012). Similarly, Titaley et al. (2010:48) also confirmed that in Indonesia, women still prefer to use traditional birth attendants and home deliveries despite the availability of registered midwives in the village. The choice of care is determined by the women's socio-economic status, educational level and cultural beliefs (Van Wyk 2005). However, even those women who choose to deliver at a hospital or clinic are discharged by midwives within six hours after normal delivery if the condition of both the mother and her baby are satisfactory (Department of Health 2007). As a result, postnatal care is rendered at home by the traditional birth attendants. The traditional birth attendants employ various indigenous practices during the care of postnatal patients (Ngunyulu & Mulaudzi 2009), which include advice to delay resumption of sexual relations post-delivery, with the intention of child spacing and to ensure complete recovery of a postnatal woman before the next pregnancy. During the postnatal period, women and babies are at risk and vulnerable to serious complications and even death due to postpartum haemorrhages and HIV-related infections, resulting in increasing numbers of maternal and neonatal deaths. Hence, the country is struggling to achieve Millennium Development Goals (MDGs) 4 and 5 (WHO 2013).

To address this challenge, the standard of postnatal care should be improved through the provision of comprehensive postnatal care services that will enable registered midwives and traditional birth attendants to work as a team and identify postnatal care practices that are a threat to the health of postnatal women and babies (South African Nursing Council 1985: 6[2]e). Identifying and addressing these postnatal care practices that are a threat to the health of women and babies might improve the quality of postnatal care, which is essential for health promotion and prevention of maternal and neonatal morbidity and mortality. To be able to identify these threats midwives and traditional birth attendants require knowledge of the indigenous and Western postnatal care practices. A study of this nature is essential to close the existing gap between the registered midwives and the traditional birth attendants during the provision of postnatal care. A similar study of an intervention involving traditional birth attendants in addressing the challenge of perinatal and maternal mortality was conducted in Pakistan. The study findings confirmed that involving traditional birth attendants in maternity care resulted in a 30% reduction of perinatal and maternal mortality (Jokhio, Winter & Cheng 2005).

The purpose of the study was to explore and describe postnatal care practices, with the intention to compare the similarities and the differences between indigenous and Western postnatal care practices. Knowledge gained from this study might enhance the involvement of traditional birth attendants as a strategy to improve the standard of postnatal care, aimed at the prevention of avoidable postnatal complications.

## Problem statement

The neonatal and maternal mortality in South Africa is high, and many lives are lost during the postnatal period, because women and their babies are at risk of complications resulting from postpartum haemorrhage (14.1%), Sepsis (9.1%) and HIV- and AIDS-related infections (40.5%; Department of Health 2012). To address this challenge, postnatal care should be rendered by traditional birth attendants and registered midwives who are knowledgeable about the indigenous and Western postnatal care practices. Knowledge of postnatal care practices might enable midwives and traditional birth attendants to identify practices that are a threat to the health of postnatal women and their babies. Identification of threats might motivate midwives and traditional birth attendants to work together to address those threats with the aim of promoting the health of postnatal women and their babies, and preventing postnatal complications leading to deaths and disabilities. To date, registered midwives and traditional birth attendants have not been working collaboratively during the provision of postnatal care, resulting in inadequate knowledge regarding postnatal care practices.

## Objective of the study

The overall aim of the study was to explore and describe postnatal care practices with the intention to compare indigenous and western postnatal care practices. The purpose was to empower registered midwives and traditional birth attendants with knowledge of postnatal care practices and to improve the quality of postnatal care.

## Significance of the study

The study findings might close a gap in the existing knowledge of traditional birth attendants and that of registered midwives regarding indigenous and Western postnatal care practices. Closing the knowledge gap could result in provision of comprehensive postnatal care by registered midwives and traditional birth attendants. Knowledge gained from this study might empower registered midwives and traditional birth attendants by giving them the skills and ability to identify the postnatal care practices that are a threat to the health of postnatal women and their babies. Identification of threats in postnatal care practices might encourage registered midwives and traditional birth attendants to work together to address those threats comprehensively with the aim of promoting the health of postnatal women and their babies. A comprehensive approach to addressing the threats by registered midwives and traditional birth attendants might improve the standard of postnatal care, prevent complications and reduce neonatal and maternal morbidity and mortality. Consequently, MDGs 4 and 5 might be achieved.

## Research questions

For the purpose of the study, the following questions guided the researchers throughout the study:

- What are the indigenous practices used by the traditional birth attendants during the provision of postnatal care?
- What are the Western practices used by registered midwives during the provision of postnatal care?
- What are the similarities and differences between the indigenous and Western postnatal care practices?

## Objectives of the study

The objectives of the study are outlined as:

- To explore and describe indigenous postnatal care practices.
- To explore and describe Western postnatal care practices.
- To compare the similarities and differences between indigenous and Western postnatal care practices.

## Definition of concepts

*Postnatal care* is defined by Fraser, Cooper and Nolte (2010:651) as the care that is provided to the mother and the newborn infant immediately after the expulsion of the placenta and membranes, and continues until six weeks after delivery. Fraser et al. (2010:652) also indicates that during this time, the woman enters a period of physical, psychological and emotional recuperation. In this study, postnatal care means the care that is provided for six weeks to postnatal women and their newborn infants, after home delivery or after discharge from the hospital or clinic.

Traditional birth attendants are defined by the WHO (1998:2) as ‘traditional, independent (of the health system), non-formally trained and community based providers of care during pregnancy, childbirth and the postnatal period’. In this study, the traditional birth attendants are grandmothers, who are recognised, trusted and selected by the community leaders as people who are knowledgeable and responsible for taking care of women during pregnancy, labour, delivery and postnatal.

*A registered midwife* is defined by the International Confederation of Midwives Council (ICM 2011:1) as a person who has successfully completed a midwifery education programme that is duly recognised in the country where it is located and that is based on the ICM Essential Competencies for Basic Midwifery Practice and the framework of the ICM Global Standards for Midwifery Education; who has the acquired the requisite qualifications to be registered and/or legally licensed to practice midwifery and use the title ‘midwife’, and who demonstrates competency in the practice of midwifery. In this study, a registered midwife is a person who takes care of women during antenatal, intra-partum and postnatal period at the clinic and/or hospitals.

## Research methods and design

### Design

A qualitative, descriptive and exploratory research design was used to ensure that the research questions were answered effectively. Unstructured interviews were conducted with the traditional birth attendants to explore and describe the indigenous postnatal care practices. An integrated literature review was conducted to identify the Western postnatal care practices (Burns & Grove 2009). A table was used to compare the similarities and differences between the indigenous and Western postnatal care practices.

### Participants

The population for the study were traditional birth attendants responsible for taking care of women during the postnatal period. The homes of the participants were used as the setting for data collection. The participants were selected purposively since only traditional birth attendants who were responsible for taking care of postnatal patients were selected (Burns & Grove 2009). The size of the sample was determined by data saturation (Brink, Van Rensburg & Van der Walt 2009). Finally, 15 interviews were conducted with the traditional birth attendants.

### Data collection methods

The participants were interviewed individually using unstructured interviews. An audiotape was used to record the interviews during data collection. Data were collected until data saturation was reached when interviewing the twelfth traditional birth attendant. The last three were additional to confirm data saturation, as recommended by Creswell (1998:55) − in phenomenological studies it is acceptable to conduct between five to 25 interviews.

Different literature including five books, seven Department of Health guidelines including *Saving Mothers* reports, South African Nursing Council Regulations and World Health Organisation guidelines were consulted to identify and describe Western postnatal care practices. A purposive sampling method was used since only literature that has information on Western postnatal care practices were selected. A comparison was made to identify the similarities and differences between the Western and indigenous postnatal care practices.

### Data analysis

Data were analysed following the process of data analysis as indicated by Tesch (De Vos et al. 2005). Literature control was done to confirm the identified findings (De Vos et al. 2005).

Data were transcribed from the audiotape. All the related topics were grouped together and coded after referring to the original transcribed data and codes on appropriate segments of the text. After coding, related topics were categorised to reduce the total list of the identified categories.

Data were grouped into two categories: similarities between the indigenous and Western postnatal care practices, and differences between the indigenous and Western postnatal care practices. Seven sub-categories were identified and developed to substantiate each category (De Vos et al. 2005:344).

## Ethical considerations

The researcher obtained letters of approval from the ethics committees of the University of Pretoria, the Department of Health Limpopo Province and from the Chief of the selected village. The individual participants gave both verbal and written informed consent.

The researcher ensured that the participants were free from physical, psychological, emotional or spiritual harm as a result of answering questions about their personal views. To ensure freedom from exploitation, the participants were made aware that their information and participation would not be used against them. The comfort of the participants was evaluated (by asking them if they felt comfortable to talk about the indigenous practices without fear of being stigmatised) to detect the risks and benefits that could have occurred during the study.

To ensure the right to self-determination, participation in the study was voluntary without any prejudicial treatment or penalty for those who refused to participate or decided to withdraw from participation. The researcher explained the nature of the study to the participants to ensure the right to full disclosure. The participants were treated fairly and equally before, during and after the study, and the researcher adhered to the agreed times of appointments. The researcher kept in confidence all the data collected from the participants (Polit & Beck 2008).

## Trustworthiness

To ensure credibility, the researcher spent enough time with the participants by making arrangements to visit them a day before the actual date of the interview with the intention to get to know the participants and build a trusting relationship with them. She deliberately set aside all she knew regarding indigenous postnatal care practices. Data were collected until data saturation occurred, and the environment encouraged the participants to feel free to express themselves in their own language. The interviews were conducted in the participants’ homes at a time that was convenient to them (Polit & Beck 2008).

To ensure dependability, the researcher gave the collected data to the supervisor so that she could examine it officially and confirm if it was correct.

## Results

This part of the study provides detailed information about the similarities and differences between the indigenous and Western postnatal care practices. In order to obtain the information, a total of 15 interviews were conducted with traditional birth attendants from the selected village in the Mopani District, Limpopo Province. The participants were selected purposively, because the traditional birth attendants are responsible for home deliveries and postnatal care. The participants represented various ages (45–70 years) and cultural backgrounds, with Sotho, Venda and Tsonga as the dominant groups. Data were collected in Xitsonga and transcribed and translated into English. Data were analysed following the process of data analysis according to Tesch (De Vos et al. 2005). Two categories and seven sub-categories that emerged from data analysis assisted the researcher to compare the similarities and differences between indigenous and Western postnatal care practices. The two identified categories were *similarities* between indigenous and Western postnatal care practices and *differences* between indigenous and Western postnatal care practices.

### Similarities between indigenous and Western postnatal care practices

Similarities between indigenous and Western postnatal care practices emerged as a category, which is supported by the maintenance of good nutrition, the prevention of postpartum bleeding and the prevention of infection as sub-categories, as illustrated in Table 1.

**TABLE 1:** Similarities between indigenous and Western postnatal care practices.

Indigenous postnatal care practices	Western postnatal care practices
Maintenance of good nutrition ‘I prepare [*a*] special meal and serve it warm … well-balanced food to promote milk production.’ (Ngunyulu & Mulaudzi 2009) ‘I encourage the woman to breastfeed the baby for two years or longer until the baby is able to feed herself/himself.’ (Ngunyulu & Mulaudzi 2009)	Maintenance of good nutrition A well-balanced diet in a breastfeeding woman is essential for production of breast milk for infant feeding (Gilbert 2011). ‘Continue breastfeeding your baby for 2 years and longer.’ (Department of Health 2012)
Prevention of postpartum bleeding ‘Bleeding post-delivery is a normal process of cleansing the uterus in preparation for it to return to its pre-gravid state, but if it is pouring, it is a sign of danger.’ (Ngunyulu & Mulaudzi 2009)	Prevention of postpartum bleeding ‘It requires early delivery of the placenta by controlled cord traction, oxytocin 5 iu/mL intramuscularly.’ (Van der Spuy & Anthony 2009). ‘It is normal to bleed after delivery, but bleeding should be minimal, heavy bleeding is regarded as a serious complication.’ (Department of Health 2012)
Prevention of infection ‘I use new razor blades for each client to prevent cross-infection, for example, when cutting the umbilical cord and shaving the hair of the newborn baby.’ (Ngunyulu & Mulaudzi 2009) ‘It is taboo for the mother and baby to share a bathing basin; it causes purulent discharge [*from the*] baby's eyes.’ (Ngunyulu & Mulaudzi 2009)	Prevention of infection ‘Maintain strict rules of aseptic technique, for an example: using a new sterile pack for each patient, baby is wiped, bathing is prohibited. Baby basin is used for baby bath and the mother baths herself in the bathroom.’ (Nolte 2011)

#### Maintenance of good nutrition

The maintenance of good nutrition emerged as a sub-category. To maintain good nutrition for the mother and the newborn infant, the traditional birth attendants indicated that they prepare a warm special meal and give to the mother with the intention to promote milk production, which will assist the woman to breastfeed the baby for at least two years. This was evident from the following quote by a 70-year-old well-known female traditional birth attendant who is in charge of the traditional birth attendants in the selected community: ‘I do not allow her to eat cold food, because cold food prevents milk production, so she will not produce enough milk which is important for infant feeding’. Another 59-year-old female traditional birth attendant, who is responsible for providing postnatal care to women and newborn infants in that community, agreed:

‘It is a taboo for a breastfeeding mother to eat cold food; her body needs warm and well balanced food every time, because if she eats cold food, the milk will stop coming out from her breasts and the baby will not have enough milk to quench the thirst.’

It is evident from these quotes that the traditional birth attendants have adequate knowledge regarding the effect of warm and well-balanced food for the breastfeeding woman. This idea was supported by Gilbert (2011:10), who stated that the woman should be encouraged to eat a well-balanced diet and increase fluid intake to improve skin integrity, gastro-intestinal activity and the absorption of iron and minerals, as well as to reduce the potential for constipation and feelings of fatigue. Furthermore, the well-balanced diet encourages the flow of breast milk, which is essential to infant feeding. Nolte (2011:217) also stressed that in order to regain strength and energy, a breastfeeding mother needs a diet high in protein and carbohydrates for milk production. However, there is a need to advise the traditional birth attendants that cold food can also be a well-balanced and nutritious diet and does not stop milk production.

The traditional birth attendants indicated that they encourage the woman to breastfeed the baby for at least two years or more. The further indicated that it is taboo to remove the baby from the breast before the age of two years − as children that age are still too young to feed themselves, they may be subject to various illnesses:

‘[*R*]emoving the baby from the breast before the age of two years is not good for the baby because at this age the baby can’t even feed herself or himself.’ (70-year-old female traditional birth attendant in charge of the team)

‘I encourage her to breastfeed the baby for two years or more, because in my culture, taking the baby off the breast before two years of age is ‘taboo’ … breast milk helps the baby to grow and protects the baby from illnesses.’ (49-year-old female traditional birth attendant who deals with the provision of postnatal care)

These quotes confirm that the traditional birth attendants believe that it is not healthy to stop breastfeeding before the age of two years as it places the baby at risk of infectious diseases due to lowered immune system. The traditional birth attendants believe that breast milk is the natural diet for a newborn. Traditionally, the baby should be breastfed until the age of two years, and the postnatal woman is given a special diet to promote lactation and enable her to breastfeed the baby. The Department of Health (2012) statement was similar:

The postnatal mother should continue breastfeeding the baby for two years or longer, because breast milk contains all the energy, vitamins and other nutrients in the correct amount that is needed by the baby. (p. 39)

The World Health Organisation's *Baby Friendly Breastfeeding Programme* added that breastfeeding should be continued for two years or more, along with home-prepared weaning foods (Cronje, Cilliers & Pretorius 2011).

#### Prevention of postpartum bleeding

Prevention of postpartum bleeding emerged as a sub-category that is practiced similarly by the traditional birth attendants and the registered midwives. The traditional birth attendants indicated that to stop bleeding, they massage the woman, tie a cloth around the abdomen and advise the woman to lie on her tummy until bleeding stops, because they know that it is a sign of danger. The following quotes illustrate this concern:

‘Immediately after the expulsion of the placenta, I massage the woman's abdomen, tie a cloth around the abdomen and advise her to lie on her tummy until the bleeding stops.’ (56-year-old female traditional birth attendant who deals with home deliveries)

‘It is normal for the woman to bleed after delivery, because the womb must be clean where the baby was situated in preparation for the womb to return to its pre-gravid state, but once I realise that the woman is bleeding heavily with clots, I know that the life of a woman is in danger and I call the ambulance.’ (61-year-old female traditional birth attendant who deals with home deliveries)

It is evident from this quote that some traditional birth attendants do have knowledge regarding the dangers of postpartum haemorrhage. They still regard it as a natural process of cleaning the uterus where the baby was situated, but they are able to differentiate between normal and abnormal bleeding. Therefore, they are able to seek medical assistance in order to save the life of a postnatal woman. Similarly, Fraser et al. (2010:538) and Van der Spuy and Anthony (2009:96) stated that prevention of postpartum haemorrhage includes early delivery of the placenta by controlled cord traction, massaging the uterus until it contracts and the clots are expressed and the administration of oxytocin 5 iu/mL intramuscularly. According to the Department of Health (2007:63), postpartum haemorrhage is regarded as one of the big five causes of maternal mortality. The midwives are encouraged to make the woman aware that it is normal to bleed after delivery. However, bleeding should be minimal because if it is severe, it can also be a complication. If they experience heavy bleeding they should report it immediately (Department of Health 2012).

#### Prevention of infection

Prevention of infection emerged as a sub-category that supports the similarities between the indigenous and Western postnatal care practices. The traditional birth attendants indicated that traditionally each woman and her newborn baby are encouraged to bath with very warm water immediately after delivery and to continue bathing twice daily. The mother is not allowed to use the same basin as the newborn infant, because they believe that baby might develop unusual skin rashes and purulent discharges from the eyes if bathed in the mother's basin. A traditional birth assistant expressed her belief:

‘It is a ‘taboo’ to use one basin for the mother and her newborn baby, because the baby becomes contaminated by her mother's dirt, which affects the sensitive skin and eyes of the infant.’ (56-year-old female traditional birth attendant who deals with home deliveries)

Some traditional birth attendants are aware that it is not safe to use one razor for different clients. The traditional birth attendants indicated that they encourage women to bring their own new razor blades for the cutting of the umbilical cord and that each razor blade is used once and discarded to prevent cross-infection. One 70-year-old female traditional birth attendant in charge of the team said: ‘I use new razor blades to cut the umbilical cord, so I advise every woman to bring a new razor blade which has never been used.’

It is evident from these quotes that the traditional birth attendants are knowledgeable about the prevention of cross-infection between the postnatal woman and the baby, as well as between the clients themselves. Similarly, Fraser et al. (2010:536) and Nolte (2011:467) pointed out that strict rules of aseptic technique should be maintained, for example, using a new sterile pack for each patient and discouraging the sharing of basins between the mother and the baby. In addition, the Department of Health (2012:30) reiterated that the baby should be wiped quickly because lying in blood for a long period might expose the baby to infections such as HIV.

### Differences between indigenous and Western postnatal care practices

Differences between indigenous and Western postnatal care practices emerged as a category supported by the following sub-categories: importance of colostrum, exclusive breastfeeding, timing of cutting the umbilical cord and method of contraception, as illustrated in Table 2.

**TABLE 2:** Differences between indigenous and Western postnatal care practices.

Indigenous postnatal care practices	Western healthcare practices
Importance of colostrum ‘Colostrum should be expressed and thrown away because it is dangerous to the baby.’ (Ngunyulu & Mulaudzi 2009)	Importance of colostrum Colostrum protects the baby against hypoglycaemia and promotes peristalsis to get rid of thick meconium (Nolte 2011).
Exclusive breastfeeding for the first 6 months ‘I give the newborn infant light, soft porridge … as early as [*at*] birth.’ (Ngunyulu & Mulaudzi 2009)	Exclusive breastfeeding Breast milk only for the first six months (Cronje et al. 2011).
Timing of cutting the umbilical cord ‘I wait for the placenta to be expelled, then I cut the cord and wrap the baby in a blanket.’ (Ngunyulu & Mulaudzi 2009)	Timing of cutting the umbilical cord Done immediately after delivery of the baby, but before expulsion of the placenta (Fraser et al. 2010)
Methods of contraception ‘Traditionally, sexual relations should be delayed until baby is two years or older.’ (Ngunyulu & Mulaudzi 2009) ‘The husband is kept busy by moving around with other wives, to give the breastfeeding woman a chance to recover and the baby to grow.’ (Republic of South Africa 1998).	Methods of contraception No restrictions as to when the sexual relations can be commenced different methods of contraception are used to prevent pregnancy (Roux 2008; Van Wyk 2005; Nolte 2011; Department of Health 2012).

#### Importance of colostrum

The importance of colostrum emerged as the first sub-category, which indicated that traditional birth attendants initiate breastfeeding differently from the midwives. Traditionally, the baby is given time to rest before it is put to the breast, and the mother is encouraged to squeeze out the first yellow milk (colostrum) because they believe that the colostrum is dirty and is dangerous to the newborn. Traditional birth attendants recommend that colostrum should be expressed until the light milk comes out, which is traditionally believed to be a safe baby-feeding practice:

‘I advise the woman to squeeze the foremilk first before she starts breastfeeding the baby for the first time, because it is too thick and might cause diarrhoea in the baby.’ (57-year-old female traditional birth attendant who deals with postnatal care)

‘[*T*]he yellow colour of the foremilk signifies dirt that is contained within the initial breast milk which causes abdominal discomfort for the baby, so it is safe to squeeze all the yellow milk until the milk is clear before putting the baby to suck from the breast.’ (43-year-old female traditional birth attendant who deals with postnatal care)

The study confirmed that the traditional birth attendants do not have adequate knowledge regarding the natural effect of colostrum in the baby's gut. Similar findings were reported in the studies conducted in Nepal where the traditional birth attendants believed that colostrum is not good for the baby because it causes diarrhoea (Thatte et al. 2009). In contrast, the Department of Health (2012:32) states that breastfeeding should be commenced within an hour after birth and the first yellow milk, the colostrum, contains vitamins and minerals that provide a protective lining for the baby's gut and help the gut develop.

#### Exclusive breastfeeding for the first 6 months

Exclusive breastfeeding for the first 6 months emerged from this category as a practice that is applied differently by the traditional birth attendants and the registered midwives. The traditional birth attendants believe that for the baby to gain weight and be calm and quiet, it needs to be fed with a very light diet immediately after birth, because breast milk in their view is just to quench the baby's thirst and not to gratify the baby's hunger. So, traditionally the baby is fed with very light and warm soft porridge first, and then breastfed. One 57-year-old female traditional birth attendant who deals with postnatal care said: ‘I prepare very light soft porridge for the baby [*at*] birth, to keep the baby calm and make her grow faster.’

Another agreed with the practice:

‘Giving breast milk only without soft porridge causes abdominal discomfort, so the baby will cry continuously until she develops a hot body and becomes ill. So I feed my grandchild very light soft porridge every time before putting her to the mother's breast; this prevents abdominal discomfort and the baby is always calm.’ (49-year-old female traditional birth attendant who deals with the provision of postnatal care)

It is evident from these quotes that the traditional birth attendants have inadequate knowledge regarding the importance of exclusive breastfeeding and the dangers of early introduction of solids during the first six months. In contrast to this practice, the *Mother Baby Friendly Initiative* strategy recommended and encouraged exclusive breastfeeding through the introduction of rooming-in, skin-to-skin care, human milk banks and breastfeeding rooms to ensure close proximity between the mother and the infant. Rooming-in enables the baby's urge to suck and its hunger to be gratified without delay (Utoo et al. 2012). South Africa recently introduced the Tshwane Declaration in 2011, which promotes exclusive breastfeeding of infants for six months without introduction of solids and mixed feeding (Department of Health 2011).

#### Timing of the cutting of the umbilical cord

Timing of cutting the umbilical cord emerged as a sub-category that is practiced differently by the traditional birth attendants and the registered midwives. The traditional birth attendants talked about the danger of cutting the umbilical cord before the placenta is expelled, as they believe that cutting the cord before expulsion will contribute to retention of the placenta and cause bleeding, resulting in serious complications and deaths. The traditional birth attendants said:

‘I encourage the woman to push the placenta out before I cut the cord. Then I wrap the baby in a blanket and put her to rest … If you cut the umbilical cord before the placenta is expelled, the placenta will fall back into the uterus and be retained, and the woman will bleed and die.’ (47-year-old female traditional birth attendant who deals with home deliveries)

It is evident from these quotes that the traditional birth attendants are taking precautionary measures to protect the mother from postpartum bleeding and retention of the placenta by delaying cutting the cord until the placenta is expelled. Meanwhile, they are exposing the baby to cold, which might lead to hypothermia, because they wait for the placenta to be expelled before they wrap the baby in a blanket. In contrast to this practice, Nolte (2011:189) indicated that immediately after the baby is delivered, the midwife should note the delivery time, clamp the umbilical cord and cut it at least 3 cm from the umbilicus, and then wrap the baby in a warm sterile cloth. Fraser et al. (2010:531) stressed that the umbilical cord should be clamped and cut immediately after the baby is born, and the baby should be covered with a warm cloth and put in a cot bed whilst the active delivery of the placenta continues using controlled cord traction.

#### Method of contraception

Method of contraception emerged as a sub-category under the differences between the indigenous and Western postnatal care practices. This sub-category also produced the practice of delayed resumption of sexual relations. The traditional birth attendants indicated that the postnatal woman is encouraged to delay resumption of sexual relations until the baby is at least two years of age. Delayed resumption of sexual relations serves as the most suitable and reliable method of child spacing:

‘I advise her to delay resumption of sexual relations until the baby is at least two years of age; this allows her body to recover completely and her baby to grow without the fear of her becoming pregnant again.’ (69-year-old female traditional birth attendant)

Traditionally, polygamy is used as a method of family planning. For example, a man is encouraged to marry many wives so as to give the breastfeeding woman time to heal and her baby to grow until the age of two years. The husband is not allowed to have sex with a breastfeeding woman. They believe that the man will be seriously ill after sex - the testes will be swollen and he could die if not treated soon enough. It is evident from these quotes that the traditional birth attendants are concerned about the physical health of postnatal women, their husbands and their babies; hence, they take extra precautions to protect them and prevent complications. The issue of polygamy is also supported by the *South African Recognition of Customary Marriage Act*, which allows men to marry more than one wife (Republic of South Africa 1998). In contrast to the abovementioned practice, polygamy is discouraged in the Western culture. People prefer to marry one wife, there are no restrictions as to when sexual relations can be commenced and they use different family planning methods for contraception, such as hormonal, barrier methods, emergency contraception, natural family planning and sterilisation (Roux 2008; Fraser et al. 2010). According to the Department of Health (2012:35), postnatal women are advised to use contraceptive methods immediately after delivery in order to have a chance to regain their health and give them a chance to feed their children adequately.

## Discussion of results

The in-depth literature control was done under each theme and in Table 1 and 2; hence it is not repeated in the discussion of the results. The findings confirmed that there are similarities and differences between the indigenous and Western postnatal care practices. The study provides an insight into the ability of traditional birth attendants to promote the health of postnatal women and prevent complications, despite lack of training regarding postnatal care. The study findings further clarify the limitations regarding the knowledge of traditional birth attendants related to the effect of practices that are detrimental to the health of postnatal women and their babies - for example, expressing all the colostrum before putting the baby on the breast.

This part of the discussion focuses on the similarities between the indigenous and Western postnatal care practices. A well-balanced diet is regarded as an effective strategy in production of milk for infant feeding by both the traditional birth attendants and the registered midwives. Breastfeeding of the baby until the age of two years or more was recommended by the traditional birth attendants, whereas the midwives recommend ingestion of colostrum, which protects the baby against illnesses such as HIV. The study also revealed that, similar to the midwives, the traditional birth attendants do know how to differentiate between normal and abnormal postpartum bleeding and, as a result, they are able to seek medical attention before complications arise. The traditional birth attendants are knowledgeable in the prevention of infection, which is evident in their practice of using a new razor for each client and separate bathing basins for the mother and the baby.

Differences between the indigenous and Western postnatal care practices were also identified. Cutting the cord and wrapping the baby before the expulsion of the placenta is regarded as a contributory factor to placental retention by the traditional birth attendants. However, by not wrapping the baby immediately, the baby is exposed to the possibility of hypothermia. On the other hand, the midwives recommend clamping and cutting the cord immediately after the delivery of the baby. This confirms the inadequate knowledge of the traditional birth attendants regarding the effect of delayed wrapping of the baby. The traditional birth attendants recommend that the colostrum should be expressed and thrown away because they regard it as dirty, which might cause diarrhoea in the baby, whilst midwives recommend ingestion of colostrum because it helps to expel the meconium, protects the baby's gut from excoriation and protects against illnesses such as HIV. It is clear that traditional birth attendants have inadequate knowledge regarding the importance of colostrum to the health of the baby. The midwives encourage exclusive breastfeeding for the first six months without introduction of solids, whereas the traditional birth attendants believe in giving the baby a light soft porridge before putting the baby to the breast. The study shows that the traditional birth attendants do not know the effects of early introduction of solids on the health of the baby. Delayed resumption of sexual relations until the baby is two years or more and polygamous marriage were confirmed as the most effective and reliable methods of contraception for the breastfeeding women. However, polygamous marriage is discouraged in Western culture and contraception is provided on discharge to postnatal women in preparation for sexual relations without fear of becoming pregnant during the postnatal period. It is evident that the traditional birth attendants are knowledgeable about the benefits of child spacing to the health of the postnatal woman and the baby. Based on the fact that the indigenous practices are not documented in midwifery books, it is concluded that midwives have inadequate knowledge regarding indigenous postnatal care practices.

## Limitations of the study

The study was conducted in one village, which limits the comparison and generalisation of results to other settings. The inadequate knowledge of the traditional birth attendants regarding the effect of delayed wrapping of the baby, the importance of colostrum to the health of the baby and the effect of early introduction of solids on the health of the baby, was also confirmed. It was evident from literature that midwives have limited knowledge regarding indigenous postnatal care practices, which might prevent midwives from detecting practices that are a threat to the lives of postnatal women and newborn babies (Ngomane & Mulaudzi 2012:38).

## Conclusion

The purpose of the study on which this article is based was to compare indigenous and Western postnatal care practices, with the intention to describe the knowledge of traditional birth attendants and midwives regarding postnatal care. Knowledge of similarities and differences between the indigenous and Western postnatal practices amongst registered midwives and traditional birth attendants is of utmost importance in the provision of quality postnatal care. Furthermore, it will also enhance and maintain a working relationship of mutual trust between registered midwives and traditional birth attendants.

It is suggested that training of registered midwives and traditional birth attendants regarding the similarities and differences between the indigenous and Western postnatal care practices is important in empowering the two groups with culturally congruent knowledge and skills.

Knowledge gained from this study might encourage researchers to conduct further research on how to integrate indigenous practices into midwifery healthcare.

## Recommendations

- Training of traditional birth attendants regarding the importance of wrapping the baby immediately after birth, of giving colostrum and of exclusive breastfeeding to improve the quality of care during the postnatal period.
- Training of registered midwives regarding indigenous postnatal care practices, so that they are able to identify and address those practices that are detrimental to the health of women and babies.
- Further research should be conducted to explore and describe more similarities and differences between indigenous and Western postnatal care practices in other areas of South Africa.
